# Temporal Alterations of Sphingolipids in Optic Nerves After Indirect Traumatic Optic Neuropathy

**DOI:** 10.1016/j.xops.2022.100217

**Published:** 2022-09-07

**Authors:** Muhammad Z. Chauhan, Paul H. Phillips, Joseph G. Chacko, David B. Warner, Daniel Pelaez, Sanjoy K. Bhattacharya

**Affiliations:** 1 Department of Ophthalmology, Jones Eye Institute, University of Arkansas for Medical Sciences, Little Rock, Arkansas; 2 Miami Integrative Metabolomics Research Center, Bascom Palmer Eye Institute, University of Miami Miller School of Medicine, Miami, Florida; 3 Dr Nasser Al-Rashid Orbital Vision Research Center, Bascom Palmer Eye Institute, Department of Ophthalmology, University of Miami Miller School of Medicine, Miami, Florida; 4 Department of Ophthalmology, Bascom Palmer Eye Institute, University of Miami Miller School of Medicine, Miami, Florida

**Keywords:** Lipid ontology, Lipidomics, Sonication-induced optic neuropathy, Sphingolipid metabolism, Traumatic optic neuropathy, CerG1, hexosylceramide, ON, optic nerve, PC, principal component, PERG, pattern electroretinogram, RGC, retinal ganglion cell, SM, sphingomyelin, ST, sulfatide, TON, traumatic optic neuropathy

## Abstract

**Purpose:**

To identify optic nerve (ON) lipid alterations associated with sonication-induced traumatic optic neuropathy (TON).

**Design:**

Experimental study.

**Subjects:**

A mouse model of indirect TON was generated using sound energy concentrated focally at the entrance of the optic canal using a laboratory sonifier with a microtip probe.

**Methods:**

Analyses of datasets generated from high-performance liquid chromatography-electrospray tandem mass spectrometry of ONs dissected from the head of the ON to the optic chiasm at 1 day, 7 days, and 14 days postsonication compared with that in nonsonicated controls.

**Main Outcome Measures:**

Lipid abundance alterations in postsonicated ONs were evaluated using 1-way analysis of variance (false discovery rate-adjusted significant *P* value < 0.01), lipid-related gene sets, biochemical properties, and receiver operating characteristic to identify lipids associated with optic neuropathy.

**Results:**

There were 28 lipid species with significantly different abundances across the control and postsonication groups. The 2 most significantly upregulated lipids included a sphingomyelin (SM) species, SM(d40:7), and a hexosylceramide (CerG1) species, CerG1(d18:1/24:2). Hexosylceramide (d18:1/24:2) was noted to have a stepwise increasing trend from day 1 to day 14 after sonication-induced optic neuropathy. Investigation of biophysical properties showed notable enrichment of lipids with high and above-average transition temperatures at day 14 after sonication. Lipid-related gene set analysis revealed enrichment in sphingolipid and glycosphingolipid metabolic processes. The best classifier to differentiate day 14 postsonication from controls, based on area under the receiver operating characteristic curve, was CerG1(d18:1/24:2) (area under the receiver operating characteristic curve: 1).

**Conclusions:**

Temporal alterations in sphingolipid metabolism and biochemical properties were observed in the ON of mice after sonication-induced optic neuropathy, with notable elevations in sphingomyelin and hexosylceramide species. Hexosylceramide (d18:1/24:2) may be associated with damage after indirect trauma, indicating that lipid membrane abnormalities may be a mediator of pathology due to trauma.

Traumatic optic neuropathy (TON) is a sight-threatening complication from acute injury to the optic nerve (ON) caused by head, orbit, or ocular trauma. The overall incidence of TON is reported to be between 0.7% and 2.5%.^1^ Common etiologies for TON in the general population include motor vehicle accidents, assaults, and falls.^2^ Traumatic optic neuropathy has been shown to develop in 0.4% of individuals who have sustained any type of trauma.^3^ The diagnosis of TON is typically made clinically when there is evidence of vision loss co-occurring with an afferent pupillary defect that is correlated with a recent traumatic injury. Additional diagnostic parameters include defects in color vision and visual field loss. Initial visual acuity loss ranges from no light perception to 20/20 vision with peripheral vision loss. Severe initial vision loss from TON has a reported prevalence of 43% to 56%.^4^ Vision loss usually occurs immediately after the injury; however, up to 10% of patients experience delayed visual loss, often occurring in the setting of indirect TON.^5^ Although the optic disc initially seems normal in TON, disc pallor develops in 1 to 2 months.

There are 2 types of TON: direct and indirect. Direct TON occurs in the setting of penetrating or compressive injuries, where the substance of the ON is directly compromised. Direct TON causes disruption of normal tissues. In comparison, indirect TON occurs in the setting of blunt force head trauma or when ocular traumatic stress is conveyed to the ON via oculofacial soft tissues and skeleton.^6^ Indirect trauma, which is more prevalent than direct TON, causes vision loss through the compromise of ON integrity through energy absorbed at impact and transmission of forces to the intracanalicular portion of the ON.^4^

The pathophysiologic mechanisms of indirect TON remain elusive. A classic and widely cited study employing holographic interferometry on human skulls showed that frontal stress deforms the orbital roof around the optic foramen, which conducts the ON, ophthalmic artery, and sympathetic nerve fibers into the orbital cavity.^7^ It has been suggested that orbital deformation could lead to the transmission of coplanar forces to the ON and cause damage through shear stress or damage to associated vasculature.^8^ In addition, damage to the ON can occur through the amplification of propagating pressure waves by soft tissue.^9^ The pathogenesis of TON is determined by primary and secondary mechanisms. Primary injury is believed to be mostly caused by the consequences of direct nerve trauma, such as axonal shearing, whereas secondary damage is triggered by mechanisms such as neuroinflammation, oxidative stress, free radicals, and gliosis.^10^

The impact of indirect TON on the local lipidome environment of the ON is not known. In this study, we used a previously developed mouse model of indirect TON in which pulsed ultrasound is used to injure the ON as it passes through the optic canal, without the need for a penetrating injury or surgical treatment.^11^ Over a 2-week period after damage, our previous study showed that the number of retinal ganglion cells (RGCs) in the retina progressively declined. The purpose of this study is to assess the lipidomic changes of the ON over this 2-week period with an assessment of visual function as determined by pattern electroretinogram (PERG) to provide additional insights into the pathophysiologic mechanisms of indirect TON and identify potential lipids associated with sonication-induced TON.

## Methods

### Animals

All animals were treated according to the Association for Research in Vision and Ophthalmology’s statement on the use of animals in ophthalmic and vision research and were used in compliance with protocols authorized by the University of Miami’s Institutional Animal Care and Use Committee. Jackson Laboratory provided C57BL/6J mice. This dataset was created using 2-month-old mice. As previously described, a sonication-induced TON model was used.^11^^,^^12^ Briefly, TON was induced in 2-month-old C57BL/6J mice in an acoustic soundproof enclosed chamber using a Branson Digital Sonifier 450 (Branson Ultrasonics). After sonication, the mice were transferred to a fresh cage with thermal assistance until they completely recovered. At 1 day, 7 days, and 14 days after exposure, ONs were meticulously dissected beginning at the ON head and proceeding to the optic chiasm. Extraction with methyl-tert-butyl ether was then carried out as previously described.^12^^,^^13^ The samples were resuspended in 50 μl of a 1:1 mixture of chloroform and methanol and stored at a temperature of 20° C until further processing.

### PERG

Pattern electroretinogram was used to assess the electrical function of ganglion cell layers in 9 mice (3 each: naïve, 7 days, and 14 days postsonication). Detailed methods have been previously reported.^11^ Briefly, PERG signals were collected from a stainless-steel needle inserted subcutaneously into the snout. At a viewing distance of 10 cm, a visual stimulus consisting of contrast-reversing gratings was oriented approximately on the optic disc projection and was undetectable to the contralateral eye. Except for the reversal frequencies, the pattern stimuli were identical for each eye. Each mouse received 3 consecutive responses to 600 contrast reversals. The PERG responses were automatically stacked to ensure uniformity and were then averaged. The major positive (P1) and negative (N2) waves, their sum of absolute values, and the major positive wave’s peak latency (P1) were automatically evaluated and computed.

### High-Performance Liquid Chromatography, Mass Spectrometry, Lipid Identification, and Relative Quantification

A liquid chromatography-electrospray tandem mass spectrometry system and an orbitrap mass spectrometer were used to study lipids (Q-Exactive, Thermo Scientific). A Thermo Scientific Acclaim 120 C18 3-m column was used with liquid chromatography–mass spectrometry grade methanol:water 60:40 v/v with 10 mM ammonium acetate as solvent A and methanol:chloroform 60:40 v/v with 10 mM ammonium acetate as solvent B. A heated electrospray ionization source was operated at a spray voltage of 4.4 kV, heated electrospray ionization source vaporization temperature of 275° C, sheath gas pressure of 45 arbitrary units, and auxiliary gas flow of 15 arbitrary units. The ion transfer tube was maintained at 350° C. A 150 to 1500 m/z was chosen as the scan range. The gradient was run at a concentration of 35% to 100% solvent B for 13 minutes and then held at 35% solvent B for 2 minutes. After 3 minutes, the gradient was raised to 100% solvent A and maintained for 2 minutes. LipidSearch 4.1.3 was used for the analysis of raw data obtained from liquid chromatography–mass spectrometry (Thermo Scientific). The following search parameters were used: product search, precursor (5/5) ppm, threshold intensity 1.0%, and M-Score 0.0. The quantification and top rank filters were enabled, the main node filters were set to Main Isomer Peaks, and the identification (ID) quality was graded from A to D. Except for fatty esters, glycoglycerolipids, and deuterated glycerolipids, all target classes were chosen. All negative adducts were chosen, and all positive adducts were chosen except for Li^+^, (CH3CH2)3NH^+^, and (CH3)2NH2^+^.

### Data Analysis

After identifying the peaks, each sample was aligned to determine the unassigned peaks. Throughout the alignment process, lipid identification was filtered using an A to C grading system. The data were divided into 4 groups (control, 1 day, 7 days, and 14 days). The ON lipidome dataset was normalized and validated through a data transformation pipeline with 3 stages before statistical and bioinformatic analysis. The first stage took the raw data captured as input and removed incomplete data instances. The second filtered data by interquartile range. The third stage transformed (square-root) and normalized (sum) the data. These data were previously submitted in Metabolomics Workbench (ID: PR000859).^12^ Despite rendering the dataset, the data were neither analyzed nor interpreted, which has been performed here.

Data analysis was performed at Jones Eye Institute at the University of Arkansas for the Medical Sciences. Statistical analysis was performed using MetaboAnalyst 5.0^14^ and STATA 14.2 (StataCorp LP). Lipid ontologies were extracted using lipid ontology for biophysical and chemical properties and the R package Rodin for category, class, and subclass visualization and enrichment.^15^ Lipid-related gene sets were generated from Kyoto Encyclopedia of Genes and Genomes, Reactome, and Gene Ontology databases using LipidSig tool for lipid-related gene enrichment analysis.^16^

## Results

**Figure 1:**
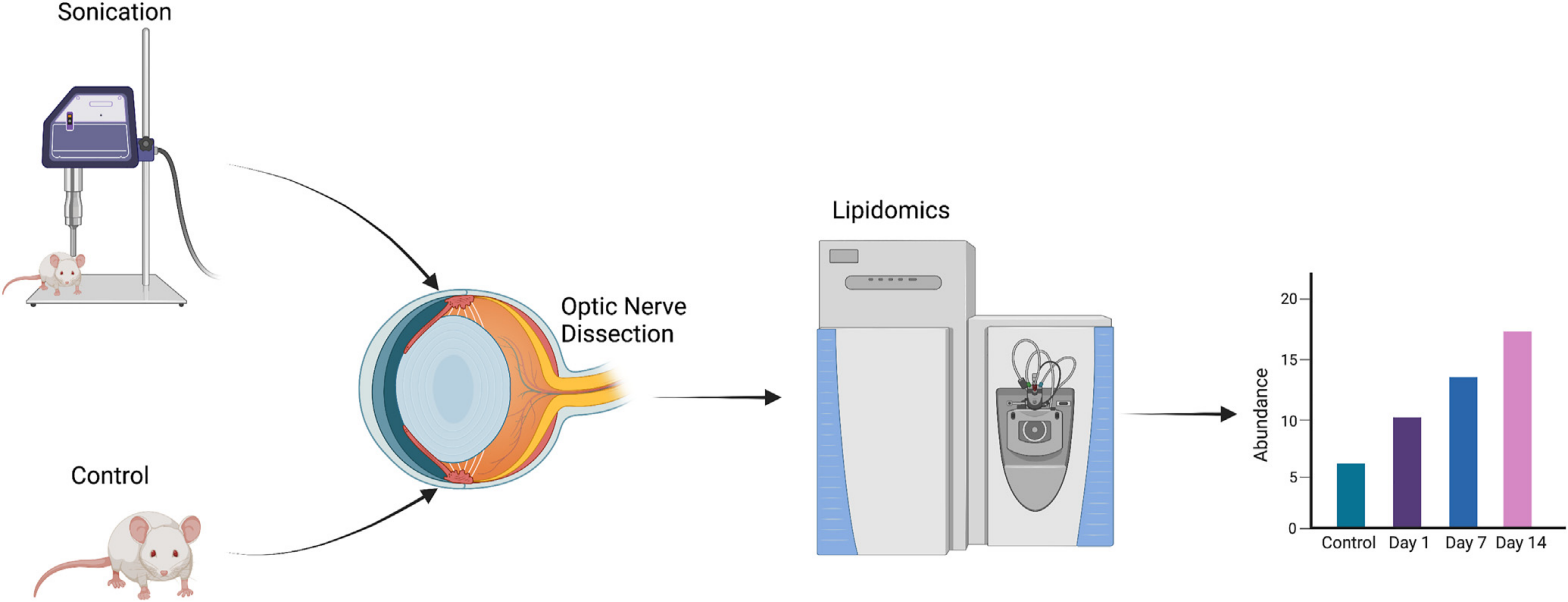
Schematic diagram of experimental design and workflow. A probe was positioned on the supraorbital rim, and 500 ms pulses with a force of 60 to 80 J were delivered. One day, 7 days, and 14 days after sonic wave exposure, mice optic nerves were collected. Mass spectrometry was used to evaluate lipid alterations in the optic nerve.

**Figure 2:**
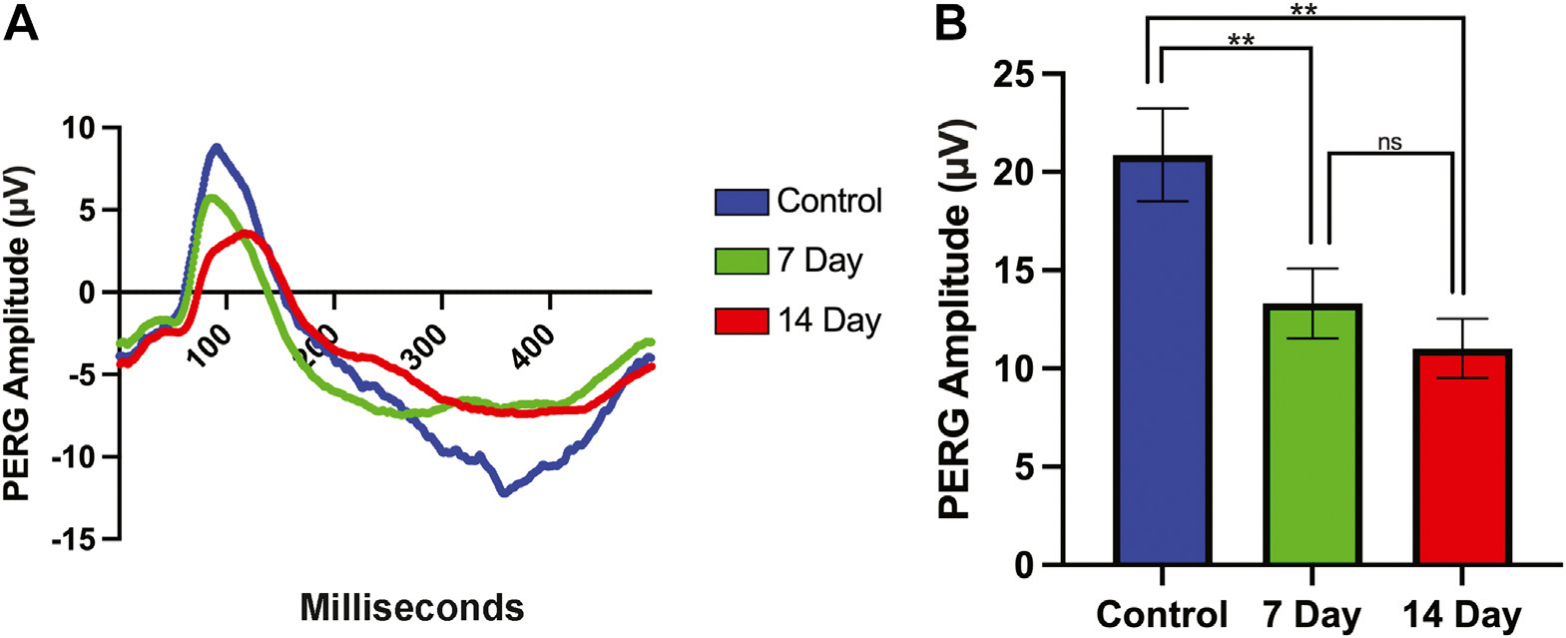
Retinal ganglion cell function after indirect traumatic optic neuropathy (TON). Pattern electroretinogram (PERGs) were recorded in controls (blue) as well as 7 days (green) and 14 days (red) after TON. **A**, Representative PERG data for the injured groups and control group were plotted after sonication-induced TON. In comparison to the control group, the injured eyes peaks are blunter in both groups, with 14-day postsonication showing the lowest peak. **B,** PERG amplitudes were compared between untreated controls and 7 days and 14 days after TON. Mean peak amplitudes were significantly lower in the 7-day and 14-day groups than in the control eyes (*P* <  0.01). Data are shown as mean ± standard deviation; ∗*P*  <  0.05; ∗∗*P* <  0.01. ns = not significant.

**Figure 3:**
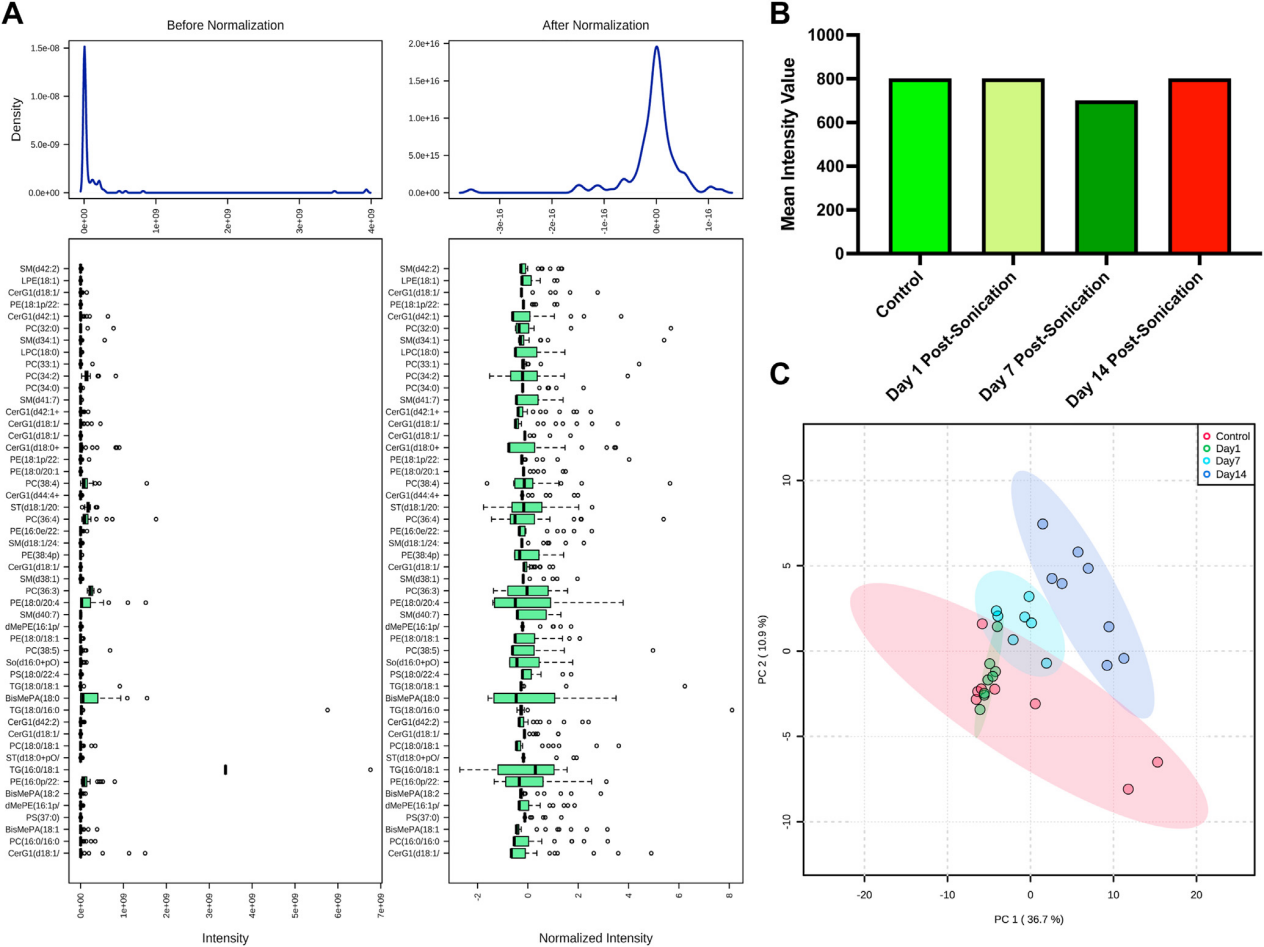
Lipidomic profiling across sonication-induced traumatic optic neuropathy groups (days 1, 7, and 14) compared with control. **A,** Box plots and kernel density plots before and after normalization. The box plots show 50 features due to space limitations. The density plots are based on all samples. Selected methods: Row-wise normalization: Normalization to constant sum; Data transformation: square-root transformed; Data scaling: auto-scaled. **B,** Total lipid (overall mean peak intensity of all lipid classes) between control and postsonication optic nerves (ONs). All groups were found to have comparable peak intensities. **C,** A 2-dimensional scores plot between the selected principal components. The explained variances are shown in brackets. Postsonication days 7 and 14 were found to be more distinct from control and day 1 postsonication. A 2-component solution explained 47.6% of the total variance observed. CerG1 = hexosylceramide; PC = phosphatidylcholine; LPE = lysophosphatidylethanolamine; PE = phosphatidylethanolamine; TG = triacylglycerol; SM = sphingomyelin. A list of the abbreviations appears in the Supplementary materials (available at www.ophthalmologyscience.org).

We performed an untargeted lipidomic analysis using high-performance liquid chromatography tandem mass spectrometry of the ON in 7 control C57BL/6J mice and 23 mice after sonication-induced trauma: 1 day (n = 8), 7 days (n = 7), and 14 days (n = 8) after exposure (Fig 1). To confirm sonication-induced functional deficits of RGCs, uninjured controls, 1-week-, and 2-week-postinjury PERG recordings were acquired and compared. In the postsonication groups, peaks were found to be blunter, with day 14 having the lowest peak compared with that at baseline (*P* < 0.01). Representative PERG results from an eye in each category (naïve, 7 days, and 14 days postsonication) revealed that the day 14 waveform was notably right-shifted compared with the uninjured control (Fig 2A). Aggregate PERG amplitudes from 9 animals (3 each: naïve, 7 days, and 14 days) were found to significantly decrease from baseline (20.87 ± 2.36 μV) to 7 days (13.31 ± 1.78 μV) and from baseline to 14 days (11.02 ± 1.51 μV) postsonication (*P* < 0.01) (Fig 2B). These findings indicate that sonication induces a consistent functional loss in the RGC layer. Lipidomic data were subsequently transformed and normalized by sum (Fig 3A). Total lipid content was comparable across the study groups (Fig 3B). We subsequently conducted a principle component analysis, which is an unsupervised learning/dimensionality reduction method to find the trends and patterns explained by the variance in the data. The principle component analysis revealed 3 clusters. Inspection of clustering showed that days 7 and 14 postsonication were more distinct from control, whereas day 1 postsonication overlapped with control, indicating that lipid species in the ONs were more distinct at days 7 and 14 postsonication compared with that in the control and at day 1 postsonication (Fig 3C). Principal component 1 (PC1) was found to explain 36.7% of the variance, and PC2 explained 10.9% of the variance. The sum of PC1 and PC2 explained 47.6% of the variance in total.

**Figure 4:**
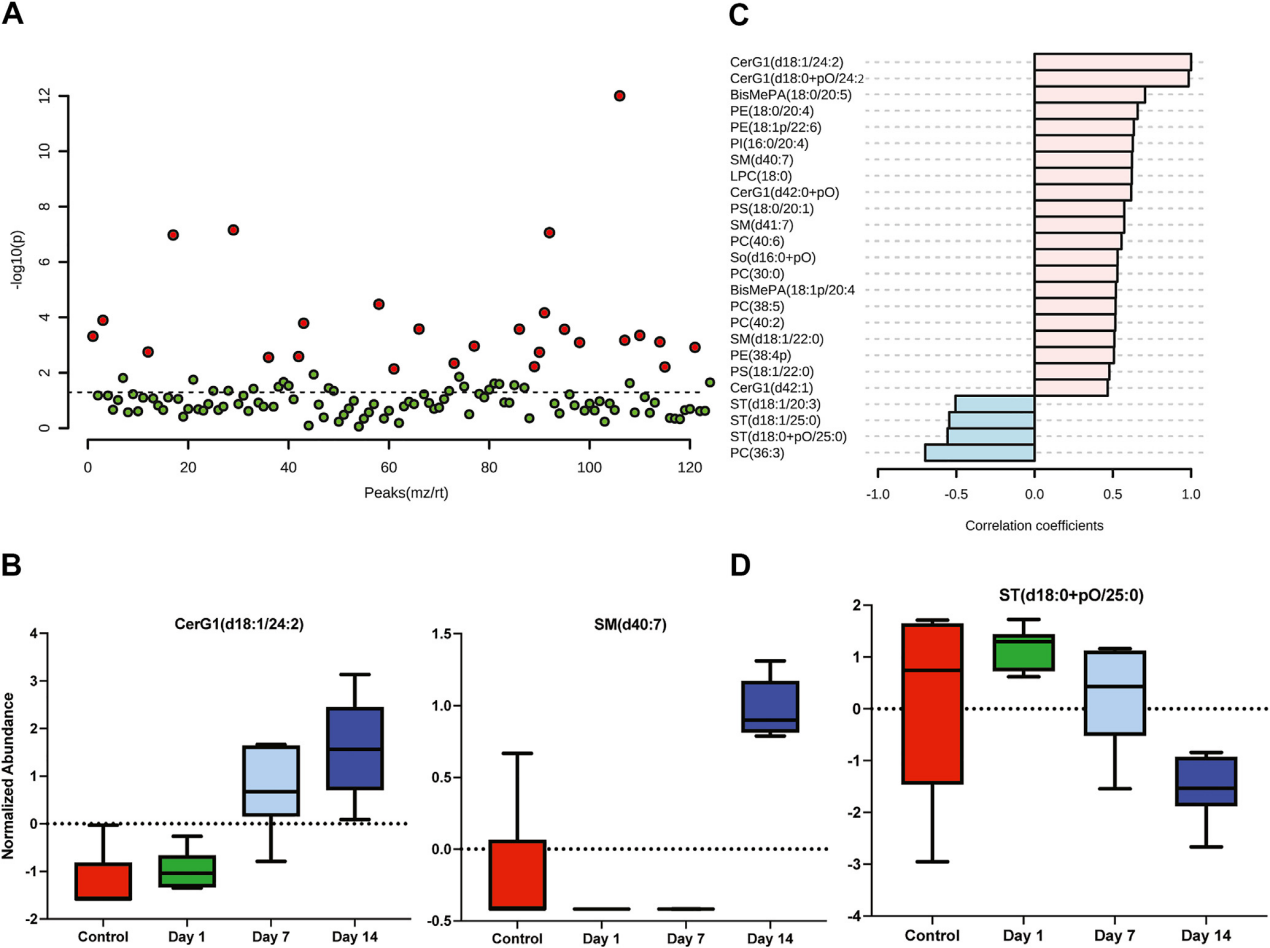
Lipidomic analysis of sonication-induced traumatic optic neuropathy (TON) optic nerves (ONs) reveals a differential abundance of lipids in a temporal pattern: differentially expressed lipids in TON ONs using mass spectrometry. Lipid abundances were compared using 1-way analysis of variance (ANOVA) analysis. **A,** Important features (lipid species) were selected by ANOVA plot with a false discovery rate (FDR)-adjusted *P* value threshold of 0.05. Red indicates values reaching the significance threshold, and green indicates low or no expression. Twenty-eight lipid species were identified with statistically significant differential abundances across the control and experimental groups. **B,** The most significant lipid species from 1-way ANOVA were found to be hexosylceramide (CerG1[d18:1/24:2]) and sphingomyelin (SM[d40:7]) (*P* < 0.001). The horizontal axis (x-axis) displays the experimental groups and the vertical axis (y-axis) displays the normalized abundances (see methods). **C,** Pearson correlation of lipid species in ONs that most significantly positively (red) and negatively (blue) correlated with a stepwise increase from control to 14 days postsonication. Graph details 21 positively correlated lipid species and 4 negatively correlated lipid species. Three sulfatide (ST) lipid species were found to significantly downtrend from day 1 to day 14 postsonication (*P* < 0.05).

A false discovery rate-adjusted 1-way analysis of variance revealed 28 lipid species with differential abundances across the control and experimental groups (Fig 4A). The 2 most significantly different lipid species across groups were found to be sphingomyelin (SM) species, SM(d40:7), and hexosylceramide (CerG1) species, CerG1(d18:1/24:2), which were both highly elevated on day 14 postsonication (Fig 4B). Notably, there was a stepwise elevation of CerG1(d18:1/24:2) from day 1 to day 14 postsonication. To determine a similar pattern across features, we performed a correlation analysis examining species correlated to CerG1(d18:1/24:2). We found that another CerG1 (CerG1(d18:01/+pO/24:2)) followed a similar pattern (Fig 4C). Several sulfatide (ST) lipid species were noted to follow the opposite trend (Fig 4C, D).

**Figure 5:**
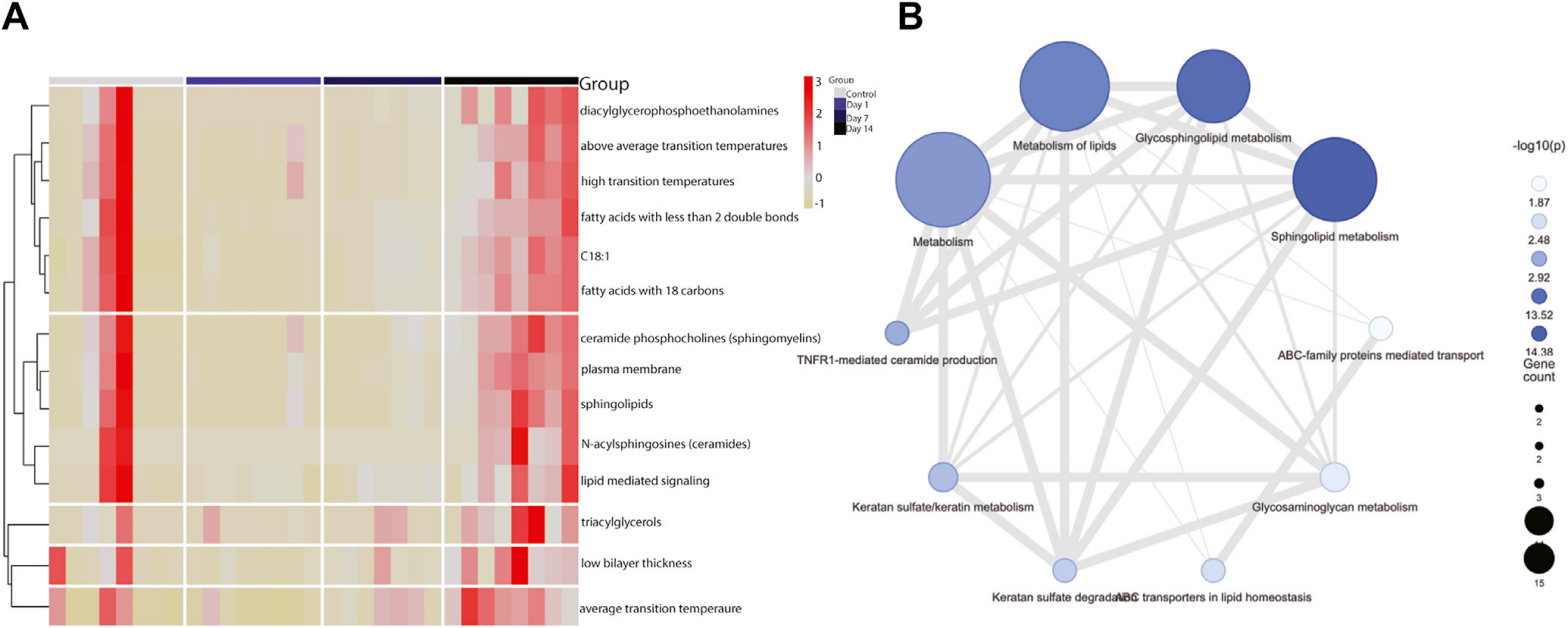
Lipidomic ontology and pathway analysis of differential abundances from analysis of variance (ANOVA). **A,** Heatmap of enriched lipid ontology terms (LION) across the experimental groups and between samples. LION analysis is based on differential abundances from 1-way ANOVA. Red indicates enrichment of term within the sample. **B,** Lipid-related gene set analysis revealed enrichment in sphingolipid and glycosphingolipid metabolic processes summarizing pathways from Kyoto Encyclopedia of Genes and Genomes (KEGG), Reactome, and Gene Ontology (GO) databases. Color of the circle is coded by *P* value, and the radius is coded by the number of interacting genes in the set.

We next investigated the changes in the biophysical and chemical properties of the post-sonication ON lipidome by examining enrichment of lipid ontology terms. A heatmap of significantly enriched lipid ontologies revealed enrichment in high and above-average transition temperatures, fatty acid chain lengths with < 2 double bonds, and fatty acid chain lengths with 18 carbons at day 14 postsonication (Fig 5A). Lipids with chain lengths with 18 carbons included species from multiple classes, including bismethyl phosphatidic acid, CerG1, phosphatidylethanolamine, phosphatidylcholine, phosphatidylserine, phosphatidylinositol, triacylglycerols, and dimethylphosphatidylethanolamine. A lipid-related gene enrichment analysis of upregulated lipid classes in postsonication day 14 ONs was subsequently run to explore significant relationships between lipid-related pathways and genes based on lipid classes. This analysis presented significantly altered pathways based on related genes of user-defined lipids.^16^ We found significant alterations in sphingolipid and glycosphingolipid metabolic processes (Fig 5B).

**Figure 6:**
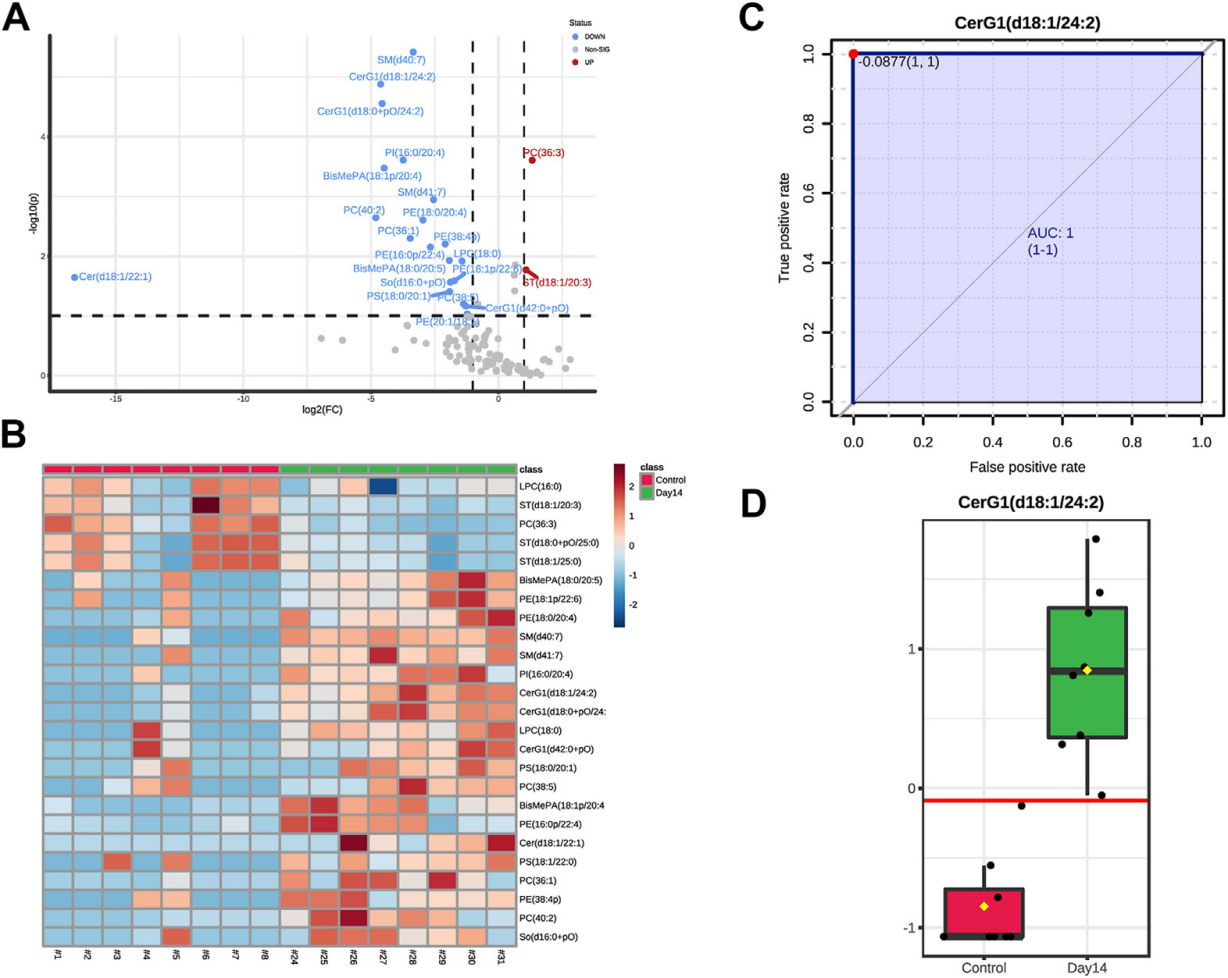
Lipid signatures of optic nerves on day 14 post-sonication. A, Lipid species fold-changes are represented as a volcano plot comparing control and 14 days postsonication. The horizontal axis (x-axis) displays the log2 fold-change value, and the vertical axis (y-axis) displays the adjusted –log10 (*P* value) from the analysis. A total of 20 lipids were downregulated in the control compared with postsonication and a total of 2 lipids were upregulated in the control (i.e., downregulated in postsonication) (*P* < 0.05). **B,** Heatmap of significantly different lipid species between control and day 14 postsonication; Euclidian distance measure and Ward clustering algorithm, features are autoscaled (red denotes positive, blue denotes negative). **C, D,** Receiver operating characteristic of hexosylceramide (CerG1[d18:1/24:2]) for distinguishing postsonication optic neuropathy from control and its abundance difference in comparison (**D**); sensitivity is shown on the y-axis and specificity on the x-axis (**C**). The area under the curve is shown in blue (area under the receiver operating characteristic curve: 1). PI = phosphatidylinositol; PE = phosphatidylethanolamine; LPC = lysophosphatidylcholine; SM = sphingomyelin; CerG1 = hexosylceramide; BisMePA= bismethylphosphatidic acid; Cer = ceramide; ST = sulfatide; So = sphingosine

Upon noticing significant alterations in the ON lipidome on day 14 postsonication, we conducted a focused analysis between the control and the day 14 group. A *t* test between the control and day 14 groups revealed 20 lipids that were significantly lower in the control group compared with that at day 14 and only 2 species that decreased postsonication. Hexosylceramide (d18:1/24:2) and SM(d40:7) were again the most significantly elevated species in the postsonication group (Fig 6A). A heatmap showing significantly different lipids between the control and day 14 groups revealed notable elevations in CerG1 and SM lipid species (Fig 6B). Classical receiving operator characteristic curves were employed to identify lipid species with the greatest ability to detect postsonication day 14. The best classifier, based on area under the receiving operator characteristic curve, was CerG1(d18:1/24:2) (area under the curve: 1) (Fig 4C). Hexosylceramide (d18:1/24:2) was significantly higher in postsonication ON (*P* < 0.001), with the ability to place 100% samples into the correct group (Fig 6D).

## Discussion

Numerous animal models for examining traumatic injury to RGCs and the ON have been developed.^10^ This is often accomplished with ON crush or transection, which are examples of direct TON where injuries directly impact the substance of the nerve. There is a need for the development of clinically relevant animal models of indirect TON injury, as this allows for the elucidation of molecular processes without confounding from tissue ultrastructure degradation that is associated with direct models. For this purpose, we previously developed an indirect model of TON in which ultrasound energy applied to the ON causes RGC depletion in mice.^11^ We previously found that 1 week after sonication, there was a significant decrease in the number of RGCs of injured eyes compared with that in control mice. In the current study, we found a continual decline of function, as analyzed by PERG, from baseline to day 14 postsonication, indicating that ganglion cell layer dysfunction is observed 2 weeks after sonication. The cause of RGC dysfunction after sonication is unknown but could result from oxidative damage from inflammatory cells.^17^ The presence of oxidative stress in TON has been demonstrated by a rise in reactive oxygen species and increases in the production of oxidative proteins after the damage.^18^ Lipid metabolites and their peroxidation products serve as indicators of oxidative stress. Lipids are soluble molecules embedded in cell membranes that perform critical tasks within the cell, such as membrane matrices, signaling, and energy storage.^19^ Lipids have several cellular functions, including lipid bilayer formation, that serve to provide membrane structure and a channel for protein transport, and act as energy depots. The lipid composition of the ON continuously changes over time and during development as a result of physiologic, pathologic, and environmental factors.^20^^,^^21^ These alterations may be mediated by disruptions in the proteome at the local or systemic level or by other mechanisms. Given the association of the lipidome changes in other optic neuropathies,^21^ we aimed to explore the lipidomic signature changes that occur during this period of functional decline.

**Figure 7:**
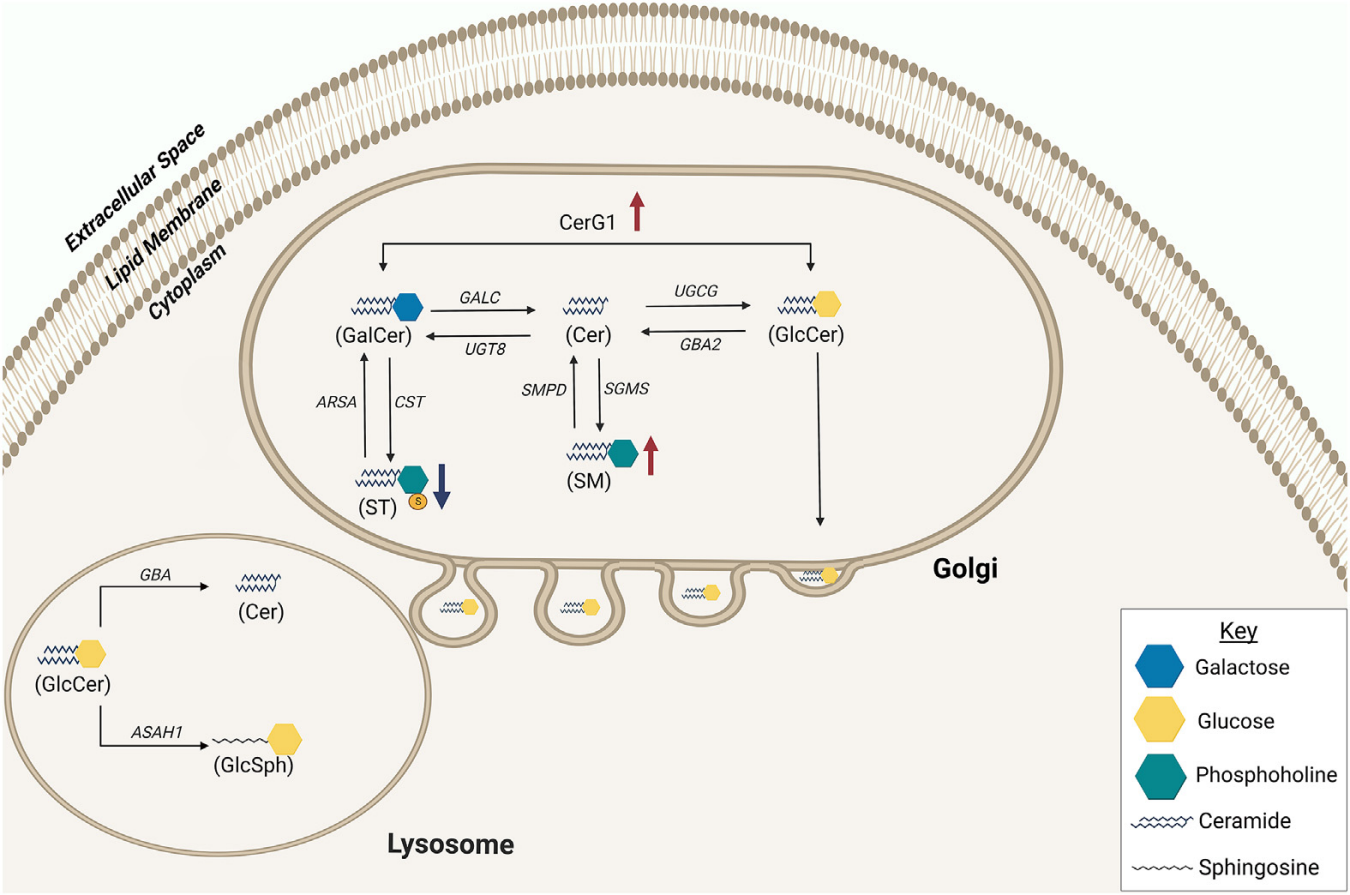
A schematic diagram of the findings in the context of the ceramide (Cer) metabolism pathway. The summary diagram depicts the Golgi body and lysosome as indicated. In the Golgi body, the ceramide could be converted into glucosylceramide (GlcCer) by *UGCG* or galactosylceramide (GalCer) by *UGT8.* Sphingomyelin (SM) is generated from Cer by *SGMS*. GalCer is the metabolic precursor to sulfatide (ST) through the action of *CST*. Glucosylceramide is released by Golgi into the cytosol that reaches the lysosomes. The Golgi releases glucosylceramide into the cytoplasm, where it reaches the lysosomes. Ceramides can be synthesized from GlcCer in lysosomes via *GBA*; alternatively, *ASAH1* can convert the pool of GlcCer to glucosylsphingosine (GluSph). CerG1 = hexosylceramide.

In this study, we found significant alterations of sphingolipids in mice after sonication-induced TON. Lipid-related gene enrichment analysis of dysregulated lipid classes found that sphingolipid and glycosphingolipid metabolism were the most altered metabolic processes in this model. We found that SM and CerG1 species significantly increased in the days after the insult. We found steady increases in a CerG1 C-18 species (d18:1/24:2) and SM(d40:7) in the days after sonication-induced TON. Notably, there was a stepwise increase in the abundance of CerG1(d18:1/24:2) from day 1 to day 14 postsonication. In addition, lipid ontology analysis showed enrichment of lipid classes with chain lengths of 18 carbons at day 14 postsonication. Sphingomyelin and CerG1 are in the sphingolipid class, which comprise a fatty acid chain joined to a long-chain sphingoid base through an amide linkage. Hexosylceramides encompass glucosylceramides and galactosylceramides. Sphingomyelin and CerG1 lipid classes are directly derived from ceramides. Sphingolipid metabolism comprises a succession of reversible events, with anabolic and catabolic mechanisms coexisting to control cellular levels of different sphingolipids. Sphingolipid metabolism begins in the endoplasmic reticulum, where the 18-carbon backbone is synthesized from nonsphingolipid precursors and the eventual formation of ceramide through the action of 4 enzyme groups.^22^ The wide family of sphingolipids, which play important roles in membrane biology and generate numerous bioactive metabolites that govern cell activity, is the result of modifications to this fundamental structure. Ceramide has low solubility in aqueous environments and is transported to the Golgi apparatus through a protein ceramide transfer protein or vesicular transport.^22^ In the Golgi, ceramides are modified to glycosphingolipids and SMs. The synthesis of glucosylceramide occurs in the Golgi from ceramide and uridine diphosphate glucose through the action of glucosylceramide synthase. Ceramide and uridine diphosphate galactose form galactosylceramide through the action of ceramide galactosyltransferase. Galactosylceramide serves as a precursor metabolite for STs; galactosylceramide sulfotransferase catalyzes the sulfation of membrane glycolipids into ST. Sphingomyelins are also generated from ceramides through the addition of a phosphocholine headgroup to ceramide by the action of sphingomyelin synthase. We found steady elevations in SM and CerG1 with depletion of ST postsonication (Fig 7). Maintaining the proper balance of these sphingolipids is critical for cell survival because an excess or depletion of any one of these lipids can be lethal to the neuron.^23^, ^24^, ^25^, ^26^

The long-chain base of sphingolipids are important molecules involved in signaling. Sphingolipids and ceramides are frequently found in the membranes of neurons, where they serve critical roles. However, accumulation of these lipids in neural tissue has been shown to impair protein transport, affect calcium homeostasis, and initiate apoptotic cascades that eventually result in neuronal cell death.^27^ For example, abnormal deoxy-long-chain base accumulation has been linked to hereditary sensory and autonomic neuropathy type 1 and taxane-induced peripheral neuropathy.^28^^,^^29^ A ceramide species fatty acyl chain can also vary in terms of chain length, saturation, and hydroxylation, with many having distinct physiologic roles.^30^ We found significant elevations in lipid species with fatty acyl chain lengths with 18 carbons, with notable elevations in CerG1 C-18 species. Additionally, we found that CerG1(d18:1/24:2) had the great ability to detect postsonication day 14. It has been shown that ceramides with a fatty acyl chain length of 18 (C-18 ceramide) inhibit cell growth by approximately 70% to 80% and induce apoptotic cell death by mitochondrial dysfunction through modulation of telomerase activity.^31^ After trauma to the central nervous system, axons need extra energy to carry out vital cellular functions, such as transport and rearrangements of cytoskeletal structure, which can be heavily disrupted by damage to mitochondria and trafficking.^32^ Another more recent study explored how the chain length of saturated fatty acids regulates mitochondrial trafficking and function in sensory neurons.^33^ The authors discovered that dorsal root ganglion neurons treated with C-18 saturated fatty acids exhibited a substantial reduction in the proportion of motile mitochondria and velocity of mitochondrial trafficking, which correlated with apoptosis. It is feasible to believe that the elevations of C-18 fatty acid chain lengths in multiple lipid classes in our study, including CerG1 and triacylglycerols, may play an important role in neuronal degeneration after indirect TON.

We also found notable declines in ST species postsonication. Sulfatides have numerous biological functions related to health and disease.^34^ Sulfatides have been shown to have immunomodulatory functions. Recently, STs were shown to ameliorate autoimmune neuritis in rats through the regulation of helper and regulatory T cells.^35^ Additionally, STs have been implicated in neurodegenerative processes, where reductions in ST levels were found in the brains of patients with Alzheimer’s disease compared with those in healthy patients.^36^ Our findings provide evidence of lipid alterations in the sphingolipid metabolic process. The increase in abundance of CerG1/SM may play a role in degeneration after ON trauma through apoptotic processes. Depletions in ST species may mediate damage through a loss of anti-inflammatory processes. These findings should be explored in future research. Additionally, combined proteomic and lipidomic studies in this model will be valuable in providing a more nuanced understanding of how sphingolipid metabolism is altered in this model.

## Conclusion

Lipidomic analysis is a powerful tool for studying the molecular basis of varying ocular pathologies and identifying novel lipid signatures for diseases. Lipid signature identification in varying diseases is important not only for clinical diagnostics but also for aiding elucidating disease mechanisms and determining new therapeutic approaches. This is a pioneering lipidomics study to identify possible lipid signatures in the ON in an indirect model of TON. We detected temporal changes in lipid species and molecular characteristics of the ON of mice after sonication-induced optic neuropathy, with considerable increases in the SM and CerG1 species. We found that elevations in CerG1(d18:1/24:2) are associated with ON damage after indirect trauma, implying that pathologic lipid membrane anomalies may play a role in disease pathogenesis.

## Supplementary Data

Supplementary Materials List of Abbreviations
